# Gender- and Age-Specific REE and REE/FFM Distributions in Healthy Chinese Adults

**DOI:** 10.3390/nu8090536

**Published:** 2016-09-01

**Authors:** Yu Cheng, Xue Yang, Li-Xin Na, Ying Li, Chang-Hao Sun

**Affiliations:** Department of Nutrition and Food Hygiene, School of Public Health, Harbin Medical University, Harbin 150086, China; chengyu-1981@126.com (Y.C.); topazbeauty@163.com (X.Y.); nalixin2003@163.com (L.-X.N.)

**Keywords:** resting energy expenditure, gender-specific, age-specific, glycolipid metabolism, eating behavior

## Abstract

Basic data on the resting energy expenditure (REE) of healthy populations are currently rare, especially for developing countries. The aims of the present study were to describe gender- and age-specific REE distributions and to evaluate the relationships among glycolipid metabolism, eating behaviors, and REE in healthy Chinese adults. This cross-sectional survey included 540 subjects (343 women and 197 men, 20–79 years old). REE was measured by indirect calorimetry and expressed as kcal/day/kg total body weight. The data were presented as the means and percentiles for REE and the REE to fat-free mass (FFM) ratio; differences were described by gender and age. Partial correlation analysis was used to analyze the correlations between REE, tertiles of REE/FFM, and glycolipid metabolism and eating behaviors. In this study, we confirmed a decline in REE with age in women (*p* = 0.000) and men (*p* = 0.000), and we found that men have a higher REE (*p* = 0.000) and lower REE/FFM (*p* = 0.021) than women. Furthermore, we observed no associations among glycolipid metabolism, eating behaviors, and REE in healthy Chinese adults. In conclusion, the results presented here may be useful to clinicians and nutritionists for comparing healthy and ill subjects and identifying changes in REE that are related to aging, malnutrition, and chronic diseases.

## 1. Introduction

Resting energy expenditure (REE) is the energy (calories) needed to maintain basic bodily functions—such as respiratory, circulatory, and nervous system functions—and is the main component of daily energy expenditure [1,2,3]. Over the past decade, scholars have demonstrated that REE is determined by multiple factors, including age [4], gender [5], ethnicity [6], body composition [7,8], and body fat distribution [9]. However, fat-free mass (FFM) is the major determinant of REE across all ages; thus, REE is often normalized for FFM via the REE/FFM ratio [10,11,12,13]. As this field of study has developed, researchers have further indicated that high and low REE values are associated with metabolic processes, with underlying metabolic disease [11], with obesity outcomes [14,15,16,17], and even with mortality [18] and multimorbidity [19,20]. On this basis, one study has suggested that REE may be used as an early biomarker of impending health deterioration [20]. Therefore, obtaining comprehensive and accurate REE data by sex, age, ethnicity, and health condition may be extremely important.

It is well-known that glycolipid metabolism and eating behaviors are associated with obesity and metabolic disease. However, it is uncertain whether glycolipid metabolism and eating behaviors are associated with REE. Recently, several studies in unhealthy populations have pointed out that higher fasting serum insulin was associated with increased REE in patients who are nondiabetic and have schizophrenia [21], and hyperglycemia and glycemic intolerance were associated with an increase in REE in patients with type 2 diabetes [22,23,24]. In addition, some recent studies showed that REE was increased by dietary protein during overfeeding [25], and high vegetable fats intake is associated with high REE in vegetarians [26]. Associations among glycolipid metabolism, eating behaviors, and REE have been reported in unhealthy subjects. However, no data are currently available on these associations in healthy subjects. Therefore, researching the relationship between glycolipid metabolism, eating behaviors, and REE in healthy subjects have a most important realistic significance in the course of medical practice for prevention of malnutrition and chronic diseases.

We performed a cross-sectional study aimed at presenting the REE and REE/FFM distributions of healthy adults by gender and age group, and at evaluating the relationships among glycolipid metabolism, eating behaviors, and REE in healthy Chinese adults.

## 2. Methods

### 2.1. Subjects

A total of 540 healthy subjects, 197 men and 343 women (from 20 to 79 years old) were included in this study. They were recruited from communities in Harbin, the largest city in northern China. Healthy subjects were defined as those who did not have a disease that might impact REE and were not taking medications known to affect REE [27]. Subjects were excluded if they had pulmonary disease, congestive heart failure, rheumatoid arthritis, cancer other than nonmelanomatous skin cancer, thyroid-stimulating hormone levels outside the normal range, or were taking medications known to affect body composition or metabolic rate (beta blockers, corticosteroids, or appetite suppressants) [28]. Each subject completed a questionnaire and underwent testing to include anthropometrics, REE assessment, and fasting blood collection. The health examination and measurements were conducted in community clinics by physicians, public health nurses, and medical technologists. The study was approved by the Ethics Committee of Harbin Medical University and conducted in accordance with the Declaration of Helsinki (chiCTR-TRC-12002829). Written informed consent was obtained from all participants.

### 2.2. Questionnaire

Each subject was interviewed privately by trained interviewers to complete a questionnaire that included items regarding name, age, gender, medical history, drug, alcohol or tobacco use, and physical activity status. Smoking was categorized as never, ≤1 cigarette/day, ≤10 cigarettes/day, ≤20 cigarettes/day, and >20 cigarettes/day. Physical activity was categorized into three groups: none, no regular strenuous physical activity; moderate, regularly engaging in strenuous physical activity at least once per week; and most active, engaging in strenuous physical activity (leisure time or occupational) at least three times per week. Alcohol consumption was calculated as the number of alcoholic drinks multiplied by the frequency.

Eating behaviors were obtained by a validated, semiquantitative food frequency questionnaire (FFQ), which was based on local eating habits. The FFQ listed 103 different foods and has been previously described [29]. Intakes of energy and macronutrients (carbohydrate, protein, fat) were calculated using the Food Nutrition Calculator (V1.60; Chinese CDC Nutrition and Food Security Institute).

### 2.3. REE Assessment

REE was measured for 30 min using indirect calorimetry (Quark PFT ergo, Cosmed Corporation, Rome, Italy), which is considered the gold standard [30]. The assessment was performed early in the morning after a fasting period of 12 h and a resting period of 15 min. During the assessment, subjects were awake and in a supine position in a quiet room. Subjects were instructed to refrain from strenuous physical activity and from taking common stimulants, such as coffee or tea, before the measurement. Indirect calorimetry was performed using a metabolic measurement system, which consisted of a facemask, a sampling pump, a computer with carbon and oxygen dioxide sensors, barometric sensors, and a turbine connecting the facemask to the computer [31]. The volume of oxygen consumption and carbon dioxide production were measured every minute. Data obtained from the last 10 min of the 30 min assessment were used to calculate the REE and respiratory quotient of each subject. The instruments were calibrated before each test.

### 2.4. Anthropometric Measurements

The same trained examiners collected the subjects’ anthropometric measurements in the morning. Before the measurements, subjects were instructed to wear light clothing and no footwear. Body height and weight were measured according to standard protocols [32], and body mass index (BMI) was calculated as weight (kg) divided by the square of the height (m). Body fat percentage was estimated by bioelectrical impedance analysis, an electric current of 0.5 mA and 50 kHz was produced by a calibrated signal generator (OMRON HBF-306; Omron, Dalian, China). In the standing position subject holds equipment in his hands with shoulders in 90 degree flexion and elbow in full extension. The subject holds the analyzer with a firm grip for approximately 7–10 s. The subject is bare-footed during the procedure. FFM and fat mass (FM) was calculated from body weight and body fat percentage [33].

### 2.5. Blood Collection and Biochemical Assessment

Antecubital venous blood samples were obtained in the morning after a 12 h overnight fast, centrifuged to obtain serum, and stored at −80 °C. Fasting blood glucose (FBG), cholesterol (TC), high-density lipoprotein cholesterol (HDL-C), low-density lipoprotein cholesterol (LDL-C), and triglycerides (TG) were determined using a Roche Modular P800 Automatic Biochemical Analyzer (Roche Diagnostics, Mannheim, Germany). In addition, serum fasting insulin concentration was measured by a Roche Elecsys 2010 Chemiluminescence Immune Analyzer (Roche Diagnostics; Laval, QC, Canada). All of these variables were measured following standard laboratory procedures, as described previously [31].

### 2.6. Statistical Analysis

The statistical analysis was performed using SPSS (version 13.01S; Beijing Stats Data Mining Co. Ltd., Beijing, China). Continuous variables were examined for normality with Kolmogorov–Smirnov tests before further statistical analysis. REE was expressed as kcal/day/kg total body weight.

Comparisons were performed after logarithmic transformation of non-normally distributed continuous variables. The demographic characteristics and glycolipid metabolism parameters of the study subjects were summarized for men and women as the means ± SD (for normally distributed continuous variables) or as the medians and interquartile ranges (for non-normally distributed continuous variables). Independent-samples *t*-tests were used to examine differences between men and women. The gender- and age-specific (20–34, 35–49, 50–64, 65–79) means, standard deviations and percentiles (5th, 10th, 25th, 50th, 75th, 90th, 95th) for REE values and REE/FFM ratios were calculated. Independent-samples *t*-tests were performed to investigate differences in the mean REE and REE/FFM ratio values of men and women. The effects of age on REE and REE/FFM were investigated by ANOVA and Tukey’s test for multiple comparisons among all age groups. Partial correlation coefficients, controlling for age, FFM, and FM, were estimated to examine the relationships between REE and glycolipid metabolism and eating behaviors. Partial correlation coefficients, controlling for age and FM, were estimated to examine the relationships between tertiles of REE/FFM and glycolipid metabolism and eating behaviors. The results were considered significant when *p* was <0.05.

## 3. Results

### 3.1. Demographic Characteristics

The demographic characteristics (age, smoking, alcohol consumption, anthropometric characteristics, education, and physical activity) of the 540 subjects are summarized in Table 1. Regarding anthropometric characteristics, men had significantly higher FFM (*p* = 0.009) and lower FM (*p* < 0.0001) than women. No significant differences were observed in BMI between women and men.

### 3.2. Glycolipid Metabolism and Eating Behaviors

Glycolipid metabolism and eating behaviors are shown in Table 2. Measures of glycolipid metabolism include FBG, fasting insulin, TC, HDL-C, LDL-C, and TG. Women tended to have higher FBG, fasting serum insulin, HDL-C, and LDL-C compared with the values observed for men. Men had higher serum TG than women. No significant differences in TC were observed by sex. Eating behaviors include energy intake, carbohydrate energy ratio, protein energy ratio, and fat energy ratio. Men tended to have higher energy intakes and carbohydrate energy ratios than women. Women had higher fat energy ratios than men. No significant differences in protein energy ratios were observed by sex.

### 3.3. Gender- and Age-Specific Absolute REE and REE/FFM Distributions

**Figure 1:**
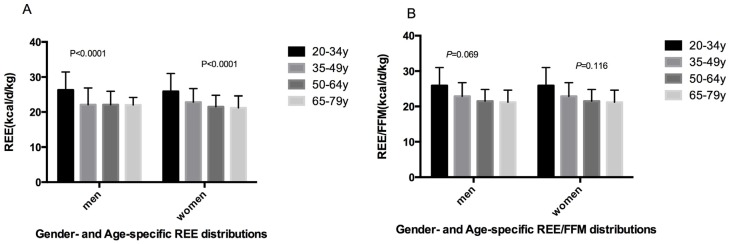
Gender- and age-specific resting energy expenditure (REE) (**A**) and REE/FFM (**B**) distributions. *p*-Value, statistically significant difference of mean values across age groups.

Table 3 and Figure 1 show results for REE and REE/FFM (means, standard deviation and percentiles) by age group for women and men. REE was higher in men than in women (*p* = 0.000). Of these men, 95% had REE values between 16.82 and 30.58 kcal/day/kg. For women, 95% had REEs between 16.19 and 28.94 kcal/day/kg. On the contrary, REE/FFM was significantly lower in men than in women (*p* = 0.021). Of these men, 95% had REE/FFM ratios between 21.77 and 41.54 kcal/day/kg. For women, 95% had REE/FFM ratios between 23.39 and 41.00 kcal/day/kg. ANOVA showed a statistically significant difference in REE by age group in men (*p* = 0.000) and women (*p* = 0.000), but no significant difference in REE/FFM by age group in men and women. Multiple comparison procedures using Tukey’s test showed that for men differences were significant between the youngest groups (20–34 year) and the other three groups (35–49, 50–64, and 65–79 years). For women, differences were significant between all groups, with the exception of the 50–64 and 65–79 years groups.

### 3.4. Partial Correlations between Glycolipid Metabolism and REE

Partial correlations between biomarkers of glycolipid metabolism and REE are shown in Table 4. The results showed no relationship between glycolipid metabolism and REE.

### 3.5. Partial Correlations between Eating Behaviors and REE

Partial correlations between eating behaviors and REE are shown in Table 5. The results showed no relationship between eating behaviors and REE.

### 3.6. Partial Correlation Coefficients between Glycolipid Metabolism and the Tertiles of REE/FFM

Partial correlations between glycolipid metabolism and the tertiles of REE/FFM are shown in Table 6. The results showed no relationship between glycolipid metabolism and the tertiles of REE/FFM.

### 3.7. Partial Correlation Coefficients between Eating Behaviors and the Tertiles of REE/FFM

Partial correlations between eating behaviors and tertiles of REE/FFM are shown in Table 7. The results showed no relationship between eating behaviors and tertiles of REE/FFM.

## 4. Discussion

This study presents an analysis of healthy 20–79 year-old adults. To the best of our knowledge, this is the first study to present REE and REE/FFM distributions as percentiles by sex and age groups, focusing on the relationships among glycolipid metabolism, eating behaviors, and REE in Chinese adults. REE was measured using indirect calorimetry, which is considered the gold standard. In this study, we confirmed a decline in absolute REE with age, and we found that men have higher absolute REE and lower REE/FFM values than women. Furthermore, we found no associations among glycolipid metabolism, eating behaviors, and REE in healthy Chinese adults.

Most previous studies of changes in REE with age have shown declines in REE, which we also found in our study [28,34,35]. Age-related decreases in REE are mainly dependent on age-related decreases in metabolically active organs’ mass [1] or body cell mass [1,36]. In addition, the decline in REE with age may not be linear in men and women [37,38]. In women, the largest decline is more pronounced in the early postmenopausal period [37,39]. In men, the largest declines occur first in early adulthood and then again in old age [39]. The same phenomenon was observed in our study: after middle age, age-related REE reductions occurred differently by gender. For women, the difference in mean values was 1.62 kg/day/kg (7.10%) between the 35–49 and 65–79 years age groups, while for men, the corresponding difference was 0.05 kg/day/kg (0.23%).

In our study, men had significantly higher FFM than women; thus, such differences in FFM may induce higher absolute REE in men. In other ethnic groups, significant differences in FFM have been found between men and women, and such differences in FFM induce variations in REE [40,41]. However, it is worth noting that in the present study, when REE was normalized for FFM, mean REE/FFM was significantly higher in women than in men. This suggests that Chinese women with less FFM have a greater REE/FFM ratio than Chinese men with more FFM. This difference in the REE/FFM ratio between the sexes has also been observed in adult participants from Milan [2]. The reasons for a difference in REE/FFM between men and women are unclear. According to the existing research, the most likely reasons for this result are that for equivalent amounts of FFM, women have a larger proportion of FFM as high-metabolic-rate tissues (e.g., brain, heart, liver, kidneys) [17], while men have a larger proportion of FFM as low-metabolic-rate tissues (e.g., skeletal muscle and bone) [10,12,42]. Metabolically active organ mass alone accounts for approximately 60% of REE in adults [43]. Skeletal muscle is the main component of FFM but only accounts for 18%–25% of REE [44]. Alternatively, other physiological factors, such as sympathetic nervous system activity and endocrine status (e.g., estrone, estradiol, progesterone, and thyroid hormone) [28], also contribute to observed population differences in REE. This analysis indicates that further research is needed to clarify the reasons for this difference between men and women.

This study found no relationships among glycolipid metabolism, eating behaviors, and REE in healthy Chinese subjects. In addition, to clarify these relationships in hyper- and hypo-metabolic subjects, we analyzed the correlation among glycolipid metabolism, eating behaviors, and the tertiles of REE/FFM further. Similarly, there was also no relationship among glycolipid metabolism, eating behaviors, and the tertiles of REE/FFM. It should be considered that these results refer predominantly to healthy Chinese subjects, and different results might be obtained for other ethnic groups or different states of health. For example, an association between blood pressure and REE has been reported in Nigerians and African Americans [45], higher fasting serum insulin is associated with increased REE in patients who are non-diabetic and have schizophrenia [21], hyperglycemia and glycemic intolerance were associated with an increase in REE in patients with type 2 diabetes [22,23,24], high vegetable fats intake is associated with high REE in vegetarians [26], and REE was increased by dietary protein during overfeeding [25]. The extant literature on the relationship among glycolipid metabolism, eating behaviors, and REE remains scarce. Therefore, longitudinal studies on different groups of people are needed to clarify the relationship among glycolipid metabolism, eating behaviors, and REE.

Although this is the first study to present the distributions of REE and the REE/FFM ratio as percentiles and assess the relationships among glycolipid metabolism, eating behaviors, and REE in Chinese subjects, it is not without limitations. First, it was performed using a self-selected sample of healthy Chinese subjects. Although this limitation is common among existing studies of REE, its results may not extend to other states of health or to other ethnic groups. Second, the sample of men in this study is small. Further elucidation of this question will require an analysis using a larger sample size. Third, given the cross-sectional nature of the study, definitive causal relationships among glycolipid metabolism, eating behaviors, and REE cannot be established. Longitudinal studies are needed to further delineate the roles of glycolipid metabolism and eating behaviors in energy expenditure.

## 5. Conclusions

In conclusion, the results presented here may be useful to clinicians and nutritionists. They provide basic data for an East Asian population for comparing healthy and ill subjects who are middle-aged or older adults and for identifying changes in REE that are related to aging, malnutrition, and chronic diseases. These data cannot be taken as universal, however, because of variation in body composition and REE among different populations due to environmental and genetic factors [6].

## Figures and Tables

**Table 1:** Demographic characteristics of the study subjects.

	Men (*n* = 197)	Women (*n* = 343)	*p*-Value
Age (year)	59.86 ± 7.57	58.77 ± 6.70	0.183
Current smokers (*n* (%))	32 (34.41)	6 (1.95)	<0.0001
Alcohol use (*n* (%))	52 (55.91)	48 (15.64)	<0.0001
BMI (kg/m)	25.04 ± 3.19	24.89 ± 3.38	0.695
FFM (kg)	52.95 ± 6.10	41.13 ± 4.72	<0.0001
FM (kg)	19.06 ± 5.40	20.78 ± 5.51	0.009
Primary education only (*n* (%))	21 (22.58)	78 (25.41)	0.343
Most active (*n* (%))	19 (20.43)	59 (19.22)	0.450

Abbreviations: BMI, body mass index; FFM, Fat-free mass; FM, fat mass.

**Table 2:** Glycolipid metabolism and eating behaviors of the study subjects.

	Men	Women	*p*-Value
*Glycolipid metabolism*			
FBG (mmol/L)	4.66 (4.24–5.75)	5.02 (4.56–5.58)	0.037
Fasting serum insulin (μU/L)	6.70 (4.40–9.65)	8.10 (5.70–12.40)	0.002
TC (mmol/L)	5.13 (4.48–5.75)	5.29 (4.70–5.81)	0.206
HDL-C (mmol/L)	1.12 (0.92–1.26)	1.31 (1.10–1.55)	<0.0001
LDL-C (mmol/L)	2.81 (2.38–3.34)	3.16 (2.58–3.74)	0.001
TG (mmol/L)	1.57 (1.08–2.92)	1.50 (0.95–2.09)	0.046
*Eating behaviors*			
Energy (kcal/day)	2640.61 ± 925.31	2137.85 ± 725.89	<0.0001
Carbohydrate (% of energy)	62.1	57.8	<0.0001
Protein (% of energy)	12.3	11.9	0.187
Fat (% of energy)	28.0	32.1	<0.0001

Abbreviations: FBG, Fasting blood glucose; TC: Total cholesterol; TG: Triglycerides; HDL-C: High-density lipoprotein cholesterol; LDL-C: Low-density lipoprotein cholesterol.

**Table 3:** Gender- and age-specific REE and REE/FFM distributions.

		*n*	Mean	SD	p5	p10	p25	p50	p75	p90	p95	*p*-Value (Age)	*p*-Value (Sex)
**REE (kcal/day/kg)**													
Men	20–34 (year)	69	26.30 ^a^	5.11	18.88	20.81	22.57	25.51	29.42	31.38	37.22	<0.0001	
	35–49 (year)	44	22.08 ^b^	4.82	13.12	15.85	18.89	22.38	25.94	29.20	29.89		
	50–64 (year)	42	22.09 ^b^	3.84	14.08	17.59	19.68	22.59	24.20	26.35	26.96		
	65–79 (year)	42	22.03 ^b^	2.16	17.69	18.67	20.80	22.10	23.76	24.61	24.93		
	All	197	23.55	4.72	16.82	18.32	20.67	23.33	25.94	29.62	30.58		
Women	20–34 (year)	20	25.89 ^a^	5.10	16.79	18.94	23.23	24.36	30.15	32.56	38.54	<0.0001	
	35–49 (year)	93	22.81 ^b^	3.86	16.45	17.36	19.83	23.22	24.93	27.47	29.56		
	50–64 (year)	130	21.47 ^c^	3.30	15.84	16.96	19.42	21.57	23.69	25.01	26.26		
	65–79 (year)	100	21.19 ^c^	3.41	16.02	17.27	19.48	20.98	23.43	25.49	26.32		
	All	343	22.01	3.78	16.19	17.28	19.77	21.87	24.18	26.37	28.94		<0.0001
**REE/FFM ratio (kcal/day/kg)**													
Men	20–34 (year)	69	33.05 ^a^	6.53	23.21	25.15	28.11	33.70	36.01	40.47	48.09	0.069	
	35–49 (year)	44	30.36 ^a^	6.63	19.68	21.06	26.57	28.94	35.36	39.34	41.94		
	50–64 (year)	42	30.73 ^a^	5.66	19.77	22.31	27.42	30.51	35.83	37.75	40.44		
	65–79 (year)	42	31.17 ^a^	4.05	23.81	25.99	27.87	31.98	33.74	35.23	37.41		
	All	197	31.56	5.99	21.77	24.51	27.38	31.60	35.31	38.31	41.54		
Women	20–34 (year)	20	34.51 ^a^	9.73	15.44	20.71	29.58	33.64	41.78	50.75	53.06	0.116	
	35–49 (year)	93	33.51 ^a^	5.91	22.51	24.52	29.53	34.08	37.71	40.20	42.70		
	50–64 (year)	130	32.56 ^a^	4.94	23.68	25.84	29.25	32.79	36.31	38.54	39.88		
	65–79 (year)	100	31.93 ^a^	5.10	23.76	25.76	28.97	31.67	35.09	38.73	40.08		
	All	343	32.75	5.65	23.39	25.57	29.26	33.15	36.67	39.19	41.00		0.021

p5, p10, p25, p50, p75, p90, and p95 correspond to the 5th, 10th, 25th, 50th, 75th, 90th and 95th percentiles, respectively. *p*-Value (sex), statistically significant difference of mean values between sexes (independent-samples *t*-test). *p*-Value (age), statistically significant difference of mean values across age groups (ANOVA). ^a,b,c^ For each measure, superscript letters next to the mean values indicate results of Tukey’s test. Means sharing the same letter indicate that values between the age groups are not significantly different from each other, whereas mean values with different superscript letters indicate that they are significantly different from each other (*p* < 0.05).

**Table 4:** Partial correlation coefficients between glycolipid metabolism and REE.

	Men		Women	
	*r*	*p*-Value	*r*	*p*-Value
FBG (mmol/L)	0.178	0.093	0.096	0.094
Fasting serum insulin (μU/L)	−0.019	0.857	0.064	0.263
TC (mmol/L)	0.180	0.089	0.085	0.138
HDL-C (mmol/L)	−0.054	0.614	−0.086	0.137
LDL-C (mmol/L)	0.006	0.952	0.015	0.795
TG (mmol/L)	0.074	0.491	0.109	0.067

Partial correlation coefficients adjusted for age, FFM and FM. Abbreviations: FBG, Fasting blood glucose; TC: Total cholesterol; TG: Triglycerides; HDL-C: High-density lipoprotein cholesterol; LDL-C: Low-density lipoprotein cholesterol.

**Table 5:** Partial correlation coefficients between eating behaviors and REE.

	Men		Women	
	*r*	*p*-Value	*r*	*p*-Value
Carbohydrate (% of energy)	−0.023	0.832	0.094	0.101
Protein (% of energy)	0.085	0.424	0.068	0.236
Fat (% of energy)	−0.027	0.804	−0.107	0.063

Partial correlation coefficients adjusted for age, FM and FFM.

**Table 6:** Partial correlation coefficients between glycolipid metabolism and the tertiles of REE/FFM.

	Tertile1 (≤29.73 kcal/day/kg)		Tertile2 (29.73–34.78)		Tertile3 (>34.78)	
	*r*	*p*-Value	*r*	*p*-Value	*r*	*p*-Value
FBG (mmol/L)	−0.022	0.807	0.096	0.124	0.076	0.387
Fasting serum insulin (μU/L)	0.005	0.955	0.028	0.748	0.024	0.789
TC (mmol/L)	−0.047	0.592	−0.053	0.546	0.030	0.732
HDL-C (mmol/L)	−0.095	0.279	−0.050	0.571	−0.023	0.797
LDL-C (mmol/L)	−0.046	0.604	0.005	0.951	0.066	0.451
TG (mmol/L)	0.064	0.470	0.045	0.612	0.006	0.950

Partial correlation coefficients adjusted for age and FM. Abbreviations: FBG, Fasting blood glucose; TC: Total cholesterol; TG: Triglycerides; HDL-C: High-density lipoprotein cholesterol; LDL-C: Low-density lipoprotein cholesterol.

**Table 7:** Partial correlation coefficients between eating behaviors and the tertiles of REE/FFM.

	Tertile1 (≤29.73 kcal/day/kg)		Tertile2 (29.73–34.78)		Tertile3 (>34.78)	
	*r*	*p*-Value	*r*	*p*-Value	*r*	*p*-Value
Carbohydrate (% of energy)	0.136	0.122	−0.031	0.725	−0.092	0.294
Protein (% of energy)	−0.050	0.570	0.015	0.862	0.084	0.341
Fat (% of energy)	−0.127	0.147	0.074	0.399	0.016	0.852

Partial correlation coefficients adjusted for age and FM.
